# Cardioprotective Role of Melatonin in Acute Myocardial Infarction

**DOI:** 10.3389/fphys.2020.00366

**Published:** 2020-04-29

**Authors:** Zhenhong Fu, Yang Jiao, Jihang Wang, Ying Zhang, Mingzhi Shen, Russel J. Reiter, Qing Xi, Yundai Chen

**Affiliations:** ^1^Department of Cardiology, The First Medical Center, Chinese PLA General Hospital, Beijing, China; ^2^Department of Cellular and Structural Biology, UT Health San Antonio, San Antonio, TX, United States; ^3^San Antonio Cellular Therapeutics Institute, Department of Biology, College of Sciences, University of Texas at San Antonio, San Antonio, TX, United States; ^4^The First Medical Center, Chinese PLA General Hospital, Beijing, China

**Keywords:** melatonin, cardioprotective, cardiomyocyte, myocardial infarction, mitochondrion

## Abstract

Melatonin is a pleiotropic, indole secreted, and synthesized by the human pineal gland. Melatonin has biological effects including anti-apoptosis, protecting mitochondria, anti-oxidation, anti-inflammation, and stimulating target cells to secrete cytokines. Its protective effect on cardiomyocytes in acute myocardial infarction (AMI) has caused widespread interest in the actions of this molecule. The effects of melatonin against oxidative stress, promoting autophagic repair of cells, regulating immune and inflammatory responses, enhancing mitochondrial function, and relieving endoplasmic reticulum stress, play crucial roles in protecting cardiomyocytes from infarction. Mitochondrial apoptosis and dysfunction are common occurrence in cardiomyocyte injury after myocardial infarction. This review focuses on the targets of melatonin in protecting cardiomyocytes in AMI, the main molecular signaling pathways that melatonin influences in its endogenous protective role in myocardial infarction, and the developmental prospect of melatonin in myocardial infarction treatment.

## Introduction

With the general improvement of the human living standard, the change of living habits and the prolongation of life span, the prevalence rate of cardiovascular diseases has risen sharply. According to *China Cardiovascular Disease Report in 2018*, the population with cardiovascular diseases in China has reached up to 290 million, and the number of patients with acute myocardial infarction (AMI) is about 2.5 million annually. AMI has become a disease seriously affecting people’s life span and quality. Despite great progress of modern medicine, science and technology, iterative new anticoagulants and antiplatelet drug, improvements in the management of AMI patients (Amanakis et al., 2019; Heusch, 2019), including the more frequent coronary reperfusion using fibrinolysis, primary percutaneous coronary intervention (PCI) or coronary artery bypass grafting (CABG), the prognosis of AMI has improved, yet, there are frequent occurrences of malignant arrhythmia events, a decline of cardiac function following AMI leading to the development of heart failure and a poor prognosis (Botker et al., 2018; Davidson et al., 2018; Joshi et al., 2019). Due to ischemia and hypoxia of the infarct area, a large number of inflammatory cells infiltrate the lesion, cardiomyocyte apoptosis is frequent and scar repair is common (Li et al., 2010; Galluzzi et al., 2018). Due to the non-regenerative feature of cardiomyocytes, irreversible cardiomyocyte apoptosis and infarction caused by acute ischemia are main factors for poor prognosis of AMI (Abukar et al., 2018; Beckendorf et al., 2018). After myocardial infarction, the myocardial tissue in the infarcted area and its adjacent non-infarcted areas undergoes apoptosis and necrosis due to ischemia, hypoxia and inflammatory response (Zhou et al., 2018c; Hu et al., 2019). Thus, the hemodynamic parameters and electrical nerve conduction of the myocardium are changed, and bring about cardiac systolic, diastolic dysfunction and dysrhythmia, and result in ventricular remodeling and electrocardiogram (ECG) changes (Zhang et al., 2014; Joshi et al., 2019). Mitochondria are organelles that generate ATP (Kowaltowski, 2019; Lee B.W.L. et al., 2019). They play a significant role in myocardial injury after myocardial infarction (Zhou et al., 2018h; Zhou et al., 2020). Stabilizing the structure and function of mitochondria effectively inhibits cardiomyocyte injury and necrosis (Audia et al., 2018; Lu J. et al., 2019). Melatonin, an indoleamine secreted by pineal gland, is a highly effective antioxidant and beneficial to many diseases including diabetes, infectious diseases, metabolic syndrome, depression, and neurodegenerative diseases (Zaouali et al., 2011; de Oliveira Junior et al., 2019). The value of melatonin in the treatment of myocardial infarction has drawn wide-spread attention in recent years. Highly compatible with mitochondrial membrane receptors, melatonin effectively reduces mitochondrial dysfunction, thus inhibiting post-myocardial infarction damage of cardiomyocytes. This review summarizes the effect of melatonin in protecting cardiomyocytes and improving prognosis after myocardial infarction by enhancing the adaptability of cardiomyocytes to ischemia and hypoxia via stabilizing mitochondrial function. We hope this introduction will be helpful to the research of melatonin in the treatment of myocardial infarction.

## Pathophysiological Changes of Heart During Myocardial Infarction

### Lipid Deposition and Atherosclerotic Plaque Formation

The direct cause of AMI is myocardial necrosis caused by prolonged ischemia and hypoxia resulting from coronary artery occlusion or spasm. The key factor leading to acute myocardial ischemia is the rupture of an atherosclerotic plaque in the coronary artery and the gradual formation of a thrombus (Zhou et al., 2018e; Bacmeister et al., 2019). The pathogenesis of atherosclerosis includes lipid deposition, inflammation, thrombosis, endothelial dysfunction, smooth muscle cell cloning and other processes (Nofe et al., 2010; Zhou et al., 2018b; Zhao J. et al., 2019). The most important risk factors are lipid metabolism disorder and endothelial cell injury. Under long-term lipid metabolism abnormality, low density lipoprotein cholesterol (LDL-C) enters intima through damaged arterial endothelium where it is converted to oxidized LDL cholesterol (Ox LDL-C), causing further damage to the intima of the coronary artery (Liu et al., 2019; Aimo et al., 2020). Ox LDL-C is engulfed by macrophages, gradually forming foam cells; there after, atherosclerotic plaques gradually develop along with the aggregation of foam cells and lipids (Abdelnaseer et al., 2016; Wu et al., 2019). If the intima of a coronary artery ruptures, the atheromatous plaque substance enters the lumen and becomes an embolus which completely occludes the blood vessel (Yuqi et al., 2015; Tian et al., 2018; Xiong et al., 2019). Thus, regulating the lipid metabolism balance, delaying the formation of atherosclerotic plaques and inhibiting plaque rupture are key measures in preventing myocardial infarction.

### Platelet Activation Aggregation and Thrombosis

When atherosclerotic plaques are formed and protrude into the lumen, local arterial stenosis causes changes in blood flow turbulence and shear force, leading to an interruption of arterial intima continuity, contraction of endothelial cells, exposure of tissues such as subendothelial collagen, and activation of platelets; this causes further adhesion and aggregation of platelets on the arterial intima, and finally formation of atherosclerotic thrombotic disease (Cohen et al., 2019; Trindade et al., 2019). Reports show that the mural thrombosis plays a vital role in pathophysiological changes of myocardial infarction progression (Hu S.Y. et al., 2017; Zhou et al., 2017b). At present, there are three types of antiplatelet drugs used in the clinic: (1) cyclooxygenase-1 (COX-1) inhibitor: aspirin. (2) ADP receptor antagonists: clopidogrel, prasugrel, ticagrelor. (3) platelet glycoprotein IIb/IIIa (GP IIb/IIIa) receptor antagonists: tirofiban, etibadin, abciximab. Among these, aspirin is a first-line antiplatelet drug and can irreversibly inactivate COX-1, an enzyme expressed in platelets, which moderates the synthesis of thromboxane; thromboxane is a potent platelet aggregation agent (Davidson et al., 2018; Merz et al., 2018).

### Inflammatory Cell Infiltration and Inflammatory Response

The microenvironment of the infarcted cardiomyocyte is a dynamic and complex area. Inflammation and immune responses play important roles in the initiation and progression of myocardial infarction. After myocardial infarction, inflammatory cells such as neutrophils, monocytes, and macrophages are activated, causing their number to increase sharply (Basalay et al., 2018; Wang Z. et al., 2019; Faghfouri et al., 2020). A large amount of inflammatory cytokines are released and move toward the injured area, and the permeability of endothelial cells increases, which results in the infiltration of inflammatory cells (Chen et al., 2016; Thieltges et al., 2018). Cytokine release and the inflammatory response are important conditions for tissue healing after myocardial infarction, but an excessive inflammatory response leads to myocardial tissue remodeling, activating apoptosis signals in cardiomyocytes with destruction of the integrity of extracellular matrix, which is not conducive to the survival of cardiomyocytes and the recovery of cardiac function (Nahrendorf et al., 2010; Krause et al., 2018). Apoptosis and the inflammatory response are initiated by myocardial tissue injury in the ischemic area. Specific cytokines and mediators including interleukin-1 (IL-1), IL-6, nuclear transcription factor kB (NF-κB) p65, NF-κB p50, Toll-like receptor 4(TLR4), Tenascin-C (TNC) and other inflammatory response regulatory proteins, as well as apoptosis regulatory proteins caspase-3, Bcl-2, etc. are produced. Excessive inflammatory response and fiber proliferation lead to ventricular remodeling (Nokik et al., 2017; Crist et al., 2018; Bocci et al., 2019; Chen M. et al., 2019). TLRs are a major reaction pathway of the inflammatory response after myocardial infarction. Recognition receptors are expressed by inflammatory cells and recognize the danger signal released by injured cells (Jager and Hoefer, 2019; Sircana et al., 2019; Yang M.Y. et al., 2019). Studies have shown that adjusting the activity of TLRs enhances the positive effect of the inflammatory response on myocardial tissue healing and limits damage to tissue, thus providing a new therapeutic process for promoting myocardial tissue recovery after myocardial infarction (Dominguez-Rodriguez et al., 2010b; Cao et al., 2019). Protein kinase D1 (PKD1) mediates the processes of myocardial remodeling, angiogenesis and myocardial contraction (Ren, 2016; Serocki et al., 2018), but the mechanism of PKD1 in the processes of the inflammatory response and cell injury in the microenvironment after myocardial infarction has not been determined.

### Cardiomyocyte Death

Cardiomyocyte injury following AMI is a very complex with multi-linked pathophysiological processes, including not only an inflammatory response, immune response, and cell signal transduction, but also complex processes such as apoptosis, necrosis, autophagy, and mitochondrial dysfunction (Westman et al., 2016; Hockings et al., 2018; Heckmann et al., 2019). Immunological studies show that cardiomyocyte in the myocardial infarction region is seriously damaged, apoptosis is severe, the expressions of Bax and caspase-3 are significantly increased, while the expression of Bcl-2 is reduced (Jin et al., 2018). It is reported that, caspase-8 is involved in fatty acid synthase (Fas)/FasL-related death receptor pathway, caspase-9 is depended on mitochondrial damage, and caspase-12 is related to endoplasmic reticulum stress (Fuhrmann and Brune, 2017; Karwi et al., 2018; Jiang et al., 2019). These are associated with cardiomyocyte apoptosis following myocardial infarction. After AMI, the focal area mainly consists of a peripheral ischemic penumbra and inner infarct area. The ischemic penumbra is mainly composed of apoptotic cardiomyocytes, and the inner infarcted area contains numerous dead cardiomyocytes (Niccoli et al., 2016; Hadebe et al., 2018). Studies reveal that the number of necrotic cells is seven times that of apoptotic cells; thus apoptosis plays only a minor role in the progression of myocardial infarction (Adameova et al., 2016; Zhou et al., 2018a; Zhu P. et al., 2018). Mitochondrial reactive oxygen species (ROS) and xanthine oxidase activity in ischemic areas lead to oxidative stress, and the calcium-induced calcium release (CICR) leads to intracellular calcium overload (Zollbrecht et al., 2016; Espinosa-Diez et al., 2018; Graczyk-Jarzynka et al., 2019; Guidarelli et al., 2019). Oxidative stress and inflammatory response activate receptor interaction protein kinase 3 (Ripk3) via interaction with cell death receptors; the common receptors are the Fas receptor, TRAIL receptor and TNFR1 (Zhou et al., 2017e; Zhou and Toan, 2020). Ripk3 destroys cell membrane integrity by mediating cell membrane-related chromosome translocation, resulting in cell necrosis (Kohlstedt et al., 2018; Zhong et al., 2019).

### Mitochondrial Dysfunction and Mitophagy

As organelles with important functions in eukaryotic cells, mitochondria mainly generate ATP, moderating apoptosis and oxidative stress reactions and exhibit dynamic changes. Their development and degradation, fusion and fission, and their quantity and morphology are precisely regulated and controlled based on changes in the environment where the cells are located (Zhou et al., 2017a,f; Chen S. et al., 2019; Mayorov et al., 2019; Simula et al., 2019). Mitochondrial dynamics include mitochondrial fission, fusion and mitophagy. Mitochondrial fission aggravates the damage to mitochondrial structure and function by inducing mitochondrial fragmentation. The fission process is considered as an initiation event for mitochondrial apoptosis (Zhou et al., 2017c; Xu Z. et al., 2019; Zhou et al., 2019). Mitochondrial fusion reduces mitochondrial damage and maintains function of these organelles by promoting the integration of mitochondrial fragments, enhancing the stability of mitochondrial genes, and promoting the exchange of mitochondrial contents (Filadi et al., 2018; Kanaan et al., 2018; Yao et al., 2019). As a process of organelle autophagy, mitophagy removes and digests damaged mitochondria fragment via mitochondrial degradation moderated by lysosomes (Zhou et al., 2018d, e; Breda et al., 2019; Mei et al., 2019). Mitophagy is specific and a main pathway of mitochondrial metabolism, and plays a key role in maintaining cell function stability (Liu et al., 2012; Bhandari et al., 2014; Kang et al., 2016).

When cellular damage causes mitochondrial structural dysfunction, cells firstly maintain their original structure through antioxidant factors, DNA repair, protein folding, and so on. If this first line of defense is breached, autophagy, fusion, and biogenesis of the mitochondria are activated, which is a more effective and extensive quality control system (Yang M. et al., 2019). The process of mitophagy is closely related to ubiquitin/proteasome system (UPS). First, the damaged mitochondria are ubiquitinated through modification and then recognized by ubiquitin receptors. The substrates are induced to mitophagy degradation by binding to LC3 on phages, and finally the components after degradation are released to the cytoplasm for recycling, providing nutrients and energy for cell survival (Ji and Kwon, 2017). Recent studies reveal that mitophagy participates in mitochondrial protection induced by mitochondrial fusion (Kornicka et al., 2019). Mitochondrial fusion initially promotes the integration of fragmented mitochondria, and then “cleans” mitochondria by proteolysis in lysosomes to moderately degrade damaged mitochondrial fragments, thus maintaining the stability of mitochondrial quality and quantity (Tahrir et al., 2019; Wang et al., 2020). In most cases, mitophagy can remove defective mitochondria from AMI injuries, playing a protective and adaptive role. The experimental results of Siddall et al. (2013) show that the enhancement of mitophagy activity protects cardiomyocytes by reducing the production of mitochondrial ROS and inhibiting calcium overload (Reddy et al., 2018). The role of mitophagy in myocardial infarction cell injury, however, is still controversial. However, experimental data of Yang et al. (2018) indicates that the rise of mitophagy reduces the energy supply of cells, thus aggravating cell injury (Koentges et al., 2018; Zhou et al., 2018g).

The heart has a very high demand for energy. There are a large number of mitochondria in cardiomyocytes. Their total volume of mitochondria in a cardiomyocyte accounts for approximately 22–37%. The energy is used to maintain the normal blood pumping function of the heart. The distribution and supply of energy are related closely to the functional state of mitochondria (Battogtokh et al., 2018; Joshi and Mochly-Rosen, 2018; Zhong et al., 2019). The mitochondrial membrane is a bilayer structure, with a non-specific ion channel in the inner membrane. This channel is known as the mitochondrial permeability transition pore (MPTP), which plays a critical role in maintaining Ca^2+^ dynamic balance and apoptosis (Meyer and Leuschner, 2018; Venugopal et al., 2018). The opening of MPTP leads to an increase of mitochondrial intimal permeability, resulting in imbalance of electrochemical driving force of ions inside and outside the membrane, a decrease of Na^+^-K^+^-ATP enzyme activity, and transmembrane transport barrier of sodium and potassium ions, which further leads to mitochondrial dysfunction, a drop of the mitochondrial inner membrane potential with the release of cytochrome c (Cyt c), and activation of the apoptosis program (Xiao et al., 2018; Xu T. et al., 2019). Under normal circumstances, MPTP is closed state and is activated when the concentration of oxygen-derived free radicals and Ca^2+^ is elevated (Yang et al., 2016; Xu S.F. et al., 2019). During AMI, cardiomyocytes are in a microenvironment of ischemia and hypoxia, with an extensive infiltration of inflammatory cells and oxygen-derived free radicals. Mitochondria then release apoptosis-inducing factors, Cyt c, pro-interleukin 1, and other mediators that induce apoptosis in this harsh microenvironment; this mediates the release of IL-1 through an inflammatory cascade reaction resulting in cardiomyocyte injury and necrosis (Teixeira et al., 2018; Lee E. et al., 2019). In addition, a large number of free radicals and inflammatory agents destroy the structure and function of mitochondria, causing inactivation of various enzymes, destruction of double membrane barrier and opening of the MPTP, causing mitochondrial edema, disintegration, and dysfunction of energy generation, and eventually leading to cardiomyocyte death and cardiac function damage. During the process of AMI, opening of MPTP plays an important role (Boengler et al., 2018; Schreiber et al., 2019). The cardiomyocyte injury in ischemia is reduced by inhibiting opening of the MPTP, lowering the release of Cyt c and limiting oxygen-derived free radicals and Ca^2+^ overload (Seidlmayer et al., 2015; Morell et al., 2018).

## Target the Melatonin Protects Cardiomyocytes in Acute Myocardial Infarction

### Melatonin Plays Anti-oxidative Stress Effect and Inhibits Inflammatory Response

Melatonin was originally discovered in the bovine pineal gland and was named after its ability to change pigmentation (melanin) in amphibian skin. Controlled by sympathetic nervous system, the synthesis and secretion of melatonin are in phase with the fluctuations in the light:dark cycle with little secretion during the day and high amounts of secretion at night (Perez-Gonzalez et al., 2019). Some of the biological effects of melatonin are related to its ability to effectively scavenge free radicals and enhance the activity of antioxidant enzymes; melatonin’s metabolites also exhibit high radical scavenging activity (Galano et al., 2013). Melatonin is highly potent free radical scavenger due to multiple mechanisms (Galano and Reiter, 2018). Free radicals that are eliminated include nitric oxide (NO⋅), superoxide anion radical (O_2_^–^⋅) and hydroxyl radical (OH⋅), etc. Its hydrophilicity and high lipophilicity, allows melatonin to pass through the cell membrane and the nuclear membrane easily, thus exerting strong antioxidant effects in cytoplasm and nucleus (Garcia et al., 2015). In addition to directly scavenging free radicals, melatonin also induces the expression of antioxidant enzymes to achieve indirect antioxidant effects (Kleszczynski et al., 2016). Nrf2 (NF-E2-related factor2), a transcription factor, plays a key role in cell oxidative stress response, and controls the expression of various antioxidant response genes after linking to the DNA antioxidant response element (ARE), while melatonin mainly influences the pathway through a nuclear retinoid-related orphan receptor (RZR/RORα) (Giudice et al., 2010).

Melatonin also plays an antioxidant role in coordination with reduced glutathione, nicotinamide adenine dinucleotide phosphate (NADPH), vitamin C, vitamin E, etc. (Yang C.H. et al., 2019). Its synergistic anti-inflammatory effects are mainly realized by up-regulating the activity of enzymes synthesizing such antioxidants so as to increase total content of antioxidants in the organism (Yoon et al., 2019). The anti-inflammatory mechanisms of melatonin include inhibiting the aggregation of inflammatory cells and the release of inflammatory cytokines including TNF-α, IL-1β, and IL-6 all of which are important inflammatory mediators of the inflammatory response. These inflammatory factors directly cause tissue injury and also stimulate other inflammatory cells to release inflammatory mediators, causing a chain reaction (Amin et al., 2019). Experiments show that melatonin increases the release of anti-inflammatory mediators such as IL-10 and while inhibiting the release of inflammatory mediators such as TNF-α, IL-1β, and IL-6, thus achieving an anti-inflammatory effect. In addition, animal experiments show that anti-inflammatory actives of melatonin are related to its inhibition of adhesion molecule related gene expression (Hu C. et al., 2017).

The TLRs mentioned above are the main response pathway of the inflammatory response after myocardial infarction. In a rat myocardial infarction model (Zhao Y. et al., 2019), the expression of the TLR4 signaling pathway is inhibited when melatonin is injected into the heart before ischemic injury. In addition, melatonin also blocks upstream signals (e.g., lipopolysaccharide binding protein CD14) of TLR4. This process can significantly reduce the release of inflammatory factors such as Granulocyte-Monocyte Colony-Stimulating Factor (GM-CSF), TNF-α, C-C Motif Chemokine Ligand 2 (CCL 2), IL-1β, IL-6, C-reactive protein (CRP), serum amyloid A, α-1 antitrypsin, while the content of Nrf2, IL-1α, heme oxygenase-1, and other anti-inflammatory cytokines rise significantly (Ter Horst et al., 2018; Ostjen et al., 2019; Tang et al., 2019).

Dyslipidemia is an independent risk factor for attack of coronary heart disease (CHD). It is reported that (Tengattini et al., 2008) melatonin regulates blood lipid, and reduces the Ox LDL-C, both of which are helpful for reducing the overall incidence of myocardial infarction injury. In terms of protecting vascular endothelial cells, melatonin reduces the degree of injury of endothelial cells in the process of myocardial infarction by inhibiting the activity of myosin light streptokinase, thus delaying the progression of atherosclerosis (Rezzani et al., 2013). In addition, several studies demonstrate that melatonin induces calcium overload and ROS generation in platelets, and activates caspase pathway and depolarizes mitochondrial membrane to mediate platelet inactivation, and this process also involves peroxisome proliferator-activated receptor γ(PPARγ)/ FUN14 domain containing 1 (FUNDC1) /mitophagy pathways (Zhou et al., 2017b; NaveenKumar et al., 2019). In addition to directly mediating platelet dysfunction, melatonin also down-regulates adhesion molecules and delays NO metabolism, thus indirectly inhibiting platelet aggregation (Girish et al., 2013). This antithrombotic effect reduces cardiomyocyte injury after myocardial infarction, and plays a role in protecting the myocardium (Dominguez-Rodriguez et al., 2010a). The circadian rhythm of melatonin release significantly reduces the activity of platelets at night, while the early morning with low melatonin levels is often the time for the occurrence of cardiovascular events. Therefore, supplementation of melatonin through external sources may effectively prevent cardiovascular events (Arushanian, 2013; Lansink et al., 2016).

### Melatonin Mediates Myocardial Protection Through Receptor and Non-receptor Pathways

In rat models of myocardial infarction, melatonin concentrations in plasma and left ventricle tissue increase sharply within 1 day, and mRNA levels of the MT1, a member of melatonin receptor, rise significantly after 2 weeks, indicating that melatonin may play an endogenous protective role in myocardial infarction (Sallinen et al., 2007). Melatonin has a biological role mainly by being bound to receptors including both membrane and nuclear binding sites. Among them, membrane receptors include melatonin receptor, TNF receptor and Notch receptor. Melatonin nuclear receptors are members of the retinoic acid related orphan nuclear receptor/retinoic acid Z receptor (ROR/RZR) family, including three subtypes: α, β, γ. The subtype α is referred to as novel endogenous myocardial infarction injury defense agent in new development progress. In rats that lack RORα receptors, the size of the myocardial infarct and degree of cardiac dysfunction after cell injury increases significantly (He et al., 2016). The related pathways by which melatonin executes its cardiac protective role via the receptor pathway includes the reperfusion injury salvage kinase (RISK) pathway, SAFE pathway and Notch pathway, with a complex association among the downstream signaling molecules (Botker et al., 2018; Coverstone et al., 2018; Shanmugam et al., 2019; Yarana et al., 2019).

The RISK pathway has an intracellular biological role primarily through the best known melatonin receptors including MT1, MT2, and MT3, all of which belong to G-protein coupled receptor family (GPCR). MT1 and MT2 have a high affinity with melatonin, while MT3 has a low affinity (Cho et al., 2019). Researchers have discovered a large number of MT1 and MT2 in the heart of rats, ducks, and coronary arteries of chicken and human beings, indicating that the cardiovascular system is a major target organ of melatonin (Hukic et al., 2017). A non-specific melatonin receptor antagonist Luzindole, eliminates the protective action of melatonin on cardiomyocytes, thus confirming their role in mediating the protective effect of melatonin on the heart (Pan et al., 2015). There are three downstream signal pathways of MT1/2, namely MAPK-ERK signal pathway, AMP-dependent protein kinase (AMPK) signal pathway and PI3K-Akt signal pathway. The three routes transmit MT1/2 activation signals from extracellular to intracellular level and mediate intracellular second messenger transmission. The downstream signaling molecules of the three pathways are crossed and connected. (a) The MAPK-ERK signaling pathway is mediated by MT1/2, the activation of MAPK-ERK up-regulates of the level of antioxidant factor Nrf2, and Nrf2 couples with DNA antioxidant reaction elements (ARE) to up-regulate the expressions of HO-1, NADPH, quinone oxidoreductase 1 (NQO1), and glutathione s-transferase 1 (GST1), and reduces the expressions of apoptotic proteins, p21 and p38 (Audia et al., 2018; Canugovi et al., 2019; Kim C.Y. et al., 2019; Liu et al., 2019). The activity of the voltage dependent anion channel (VDAC) and the transcription factor of IP3R-cAMP response element binding protein (CREB) are inhibited by activated extracellular signal regulating kinase (ERK), while excessive activation of VDAC and CREB leads to intracellular calcium overload and then causes mitochondrial dysfunction, eventually bringing about cardiomyocyte necrosis (Li et al., 2018; Zhu H. et al., 2018). Activation of this pathway also leads to inactivation of glycogen synthase kinase-3β (GSK-3β) (Nduhirabandi et al., 2012). The downstream effects also involve the activation of endothelial nitric oxide synthase (eNOS), PKC, and p70 ribosomal protein S6 (p70S6), and down-regulation of the expression of apoptosis related factors such as Bax, Bad, and phosphorylation of caspases (Paradies et al., 2015; Eid et al., 2018; Wang S. et al., 2019; Xu N. et al., 2019). In addition, the activation of MAPK-ERK signaling pathway directly inhibits the opening of MPTP. (b) In the AMPK signaling pathway, Nrf2 is also a downstream signaling molecule of AMPK-PKG1α pathway (Wu et al., 2018b; Lu M. et al., 2019). The AMPK pathway and MAPK-ERK pathways are interrelated through Nrf2 and have a synergistic role in antioxidative stress processes and reducing apoptosis (Yu et al., 2018). In addition, the activation of AMPK inhibits the activity of mitochondrial motility related protein Drp1, which promotes mitochondrial fission, thereby activating VDAC-HK opening and ultimately promoting the MPTP opening (Singhanat et al., 2018). SIRT1 and SIRT3 are both important downstream signaling molecules that aid melatonin in its cardioprotective role. SIRT3 is the downstream target of peroxisome proliferator-activated receptor γ co-activator 1α (PGC-1α) which is stimulated by AMPK. The effect of this pathway is to reduce the transfer of Bax to mitochondria, promote the deacetylation of mitochondrial antioxidant enzyme GPX, boost the biosynthesis of mitochondria, and enhance the activity of superoxide dismutase (SOD) (Lombard and Zwaans, 2014; Lochner et al., 2018). (c) The primary downstream molecular effect of the PI3K-Akt signaling pathway is the reduction of cellular oxidative stress. The activation of Akt promotes phosphorylation of signal transducer and activator of transcription 3 (STAT3), thereby elevating TNFα release for cardiac protection (Yu et al., 2016; Kim E.H. et al., 2019). The enhanced activity of GPX and SOD raises the level of Nrf2, thus promoting the Akt signaling pathway (Song et al., 2017; Zhang H. et al., 2019). Melatonin regulates the activity of ERK through the Akt pathway. Signaling molecules in this pathway include Zrt/Irt-like protein 1 (Zip1), brain-derived neurotrophic factor (BDNF) and PPARγ. In the nuclear receptor signaling pathway, melatonin regulates autophagy and Cyt c release through ROR α, and also enhances the expression of the myocardial sarcoplasmic reticulum Ca^2+^-ATPase (SERCA)2α, sodium-calcium exchange 1 (NCX1), Ryanodine receptor 2 (RyR2), Ca^2+^-calmodulin-dependent kinase II (CAMKII) and other protein-related genes, thus enhancing the ability of cells to process calcium ions and reduce the stress injury to and apoptosis of the cardiomyocytes (Gebhard et al., 2018; Na et al., 2019).

In addition to the conventional melatonin receptor pathway, melatonin also binds to other receptors on the cell membrane for signal transduction, including the SAFE pathway and the Notch pathway. In the SAFE pathway, melatonin plays a role in phosphorylation of JAK2-STAT3 through TNF receptor on the cell membrane. Downstream molecular effects include the promotion of expression of BCL-2, antioxidant genes, TNFα, mcl 1, FAS and the inhibition of Bax, caspase-3, Cyt c, cyclin-dependent kinase (cyclin D1), P21 and GSK-3β (Yang et al., 2013). Melatonin also directly inhibits MPTP opening through this pathway. Phosphorylation of STAT3 activates the ERK and Akt pathways, which also promote phosphorylation of STAT3. In the Notch pathway, melatonin promotes the expression of Hairy and enhancer of split 1 (Hes 1) through Notch 1-Notch Intracellular area (NICD), while Hes1 inhibits the negative regulatory effect of chromosome 10 (PTEN) on phosphatidylinositol 3-kinase (PI3K). Notch pathway also reduces the effects of cardiomyocyte apoptosis by regulating mitophagy with mitochondrial fusion related protein (Mfn2) (Pei et al., 2016).

**FIGURE 1:**
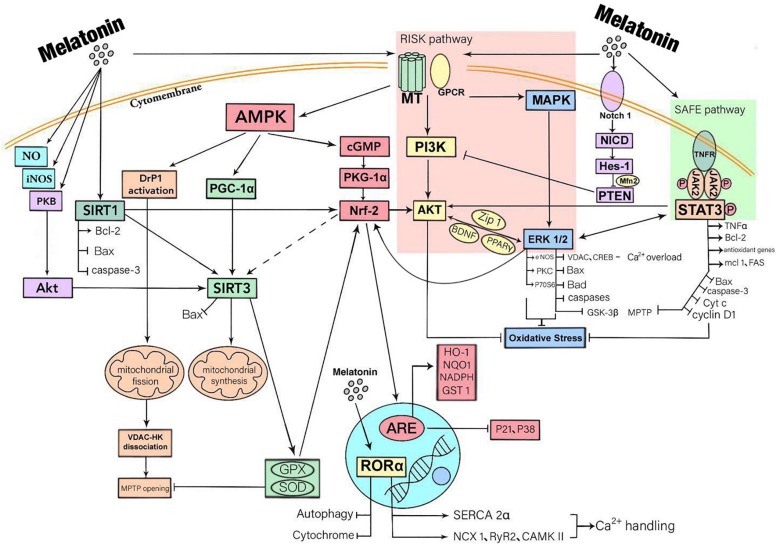
A summary of the mechanisms that myocardial protection mediated by melatonin through receptor and non-receptor pathways. Melatonin has a biological role mainly by being bound to receptors. There are three downstream signal pathways of MT1/2, namely MAPK-ERK signal pathway, AMP-dependent protein kinase (AMPK) signal pathway and PI3K-Akt signal pathway. Melatonin also activates STAT3, a signal transducer and activator of transcription factor for antioxidant enzymes, by activating SAFE pathway. Besides, melatonin activates the Notch1 pathway, thus inhibiting PIK3 function. These downstream signaling molecules are crossed and connected within the cell. In addition, melatonin also enters cells where it has direct biological effects. It promotes the release of NO, enhances the activity of iNOS and boosts the expression of SIRT3 via the activation of PKB-Akt. The ultimate effects of these responses are to reduce oxidative stress, inflammatory responses, and to protect mitochondrial function, thereby reducing cardiomyocyte apoptosis.

In addition to binding to receptors, melatonin also enters cells where it has direct biological effects (Mauriz et al., 2013). Melatonin enters the cytosol to promote the release of NO, enhances the activity of nitric oxide synthase (iNOS) and boosts the expression of SIRT3 via the activation of PKB-Akt. Activated by melatonin, SIRT1 regulates oxidative stress in cardiomyocytes, mitophagy and apoptosis by enhancing the expression of Bcl-2 and weakening Bax and caspase-3. Studies have shown that SIRT1 is an important upstream molecule of Nrf2 and can also be bound to the SIRT3 promoter to enhance the expression of SIRT3. However, whether melatonin regulates the expression of SIRT3 through the SIRT1-Nrf2 pathway requires verification (Figure 1).

### Melatonin Stabilizes the Structure and Function of Mitochondria After AMI

Mitochondria are the site of ATP and oxygen-derived free radical production, and the target of attack by various free radicals (Connolly et al., 2018; Boengler et al., 2019; Yuan et al., 2019). Melatonin protects cardiomyocytes by stabilizing the structure and function of mitochondria and regulates mitochondrial oxidative stress, raises mitochondrial antioxidant enzyme levels, restores mitochondrial energy metabolism (Soto-Heras et al., 2019), maintains mitochondrial membrane potential stability, reduces mitochondrial injury, and inhibits mitochondrial apoptosis (Yan et al., 2018) through receptors including MT1/2. In addition, mitochondrial biosynthesis, DNA homeostasis and regulation of SIRT3 system are closely related to the function of melatonin (Reiter et al., 2017; Zhou et al., 2017d). After myocardial infarction, injured tissue releases a large quantity of oxygen-derived free radicals, inflammatory mediators and other harmful substances, which cause direct injury to mitochondria (Kalkavan and Green, 2018; Riehle and Bauersachs, 2018; Nanadikar et al., 2019). Mitochondrial dysfunction aggravates cell injury, which then becomes a vicious cycle. Melatonin breaks this vicious cycle by virtue of its potent free radical scavenging ability and antioxidant effects, thus playing a protective role in myocardium such as in ischemia-reperfusion injury (Reina and Martínez, 2018). Studies show that melatonin, as a potent free radical scavenger, is abundant in mitochondria (Venegas et al., 2012). This shows that melatonin likely prevents mitochondrial injury during the oxidative stress response (Ma et al., 2016). Melatonin also up-regulates the activity of the four respiratory complexes, thus reducing electron leakage and the generation of oxygen-derived free radicals (Nair et al., 2011). Melatonin inhibits mitochondrion fission, prevents disintegration of VDAC1 and hexokinase 2 (HK2), inhibits MTPT opening, limits endothelial cell injury, improves endothelial barrier function, reduces inflammatory cell infiltration, restores eNOS content and blood flow, lowers infarct size, preserves myocardial microvasculature, and improve prognosis (Zhang and Zhang, 2014; Li et al., 2019). Based on published reports (Wang et al., 2016), activation of MT1/2 receptor strengthens AMPK signaling pathway, up-regulates optic atrophy 1 (OPA1) level and then modulates mitophagy during myocardial infarction. Mitophagy degrades injured mitochondria so as to maintain the structure and function of normal mitochondria, reduces cardiomyocyte apoptosis and lowers the degree of myocardial injury during infarction (Dominguez-Rodriguez et al., 2017a; Zhang Y. et al., 2019).

## Clinical Application and Prospect of Melatonin Use in Acute Myocardial Infarction

Due to the non-renewability of cardiomyocytes, it is essential to reduce cardiac damage and improve long-term prognosis of patients with myocardial infarction by clarifying the mechanisms of injury and necrosis of cardiomyocytes after a cardiovascular episode (Gaspar et al., 2018; Wu et al., 2018a; Heusch, 2019). In addition to the mechanisms of inflammatory cell infiltration and oxidative stress injury which have been reported many times, mitochondrial injury and apoptosis are the key initiating processes of myocardial injury following myocardial infarction (Hofmann, 2018; Villalobos et al., 2018; Wang Y. et al., 2018; Boengler et al., 2019; Phan et al., 2019). There is an urgent need to find a method which can effectively reduce mitochondrial injury and to improve cardiomyocyte function following myocardial infarction and provide a new target for clinical treatment.

As an indole and age-related molecule secreted by the human pineal gland, melatonin has remarkable functions including antioxidant, anti-apoptosis, anti-fibrosis, direct free radical scavenging, and mitochondrial protection (Jumnongprakhon et al., 2016). Melatonin receptors also play a role in reducing cardiac damage (Liu et al., 2016). In models of myocardial reperfusion injury, melatonin promotes mitochondrial fusion through AMPK/OPA1 signaling pathway and OPA1 binds with lysine 70 residues of mitophagy receptor FUNDC1 to mediate mitophagy (Del Dotto et al., 2018; Wang B. et al., 2018). Recent studies reveal that mitochondria exhibit biological rhythms, which may be regulated by the melatonin cycle. However, studies have not been conducted to clarify the role of melatonin in the mitochondrial biological clock and the myocardial protective effects that involve these rhythms. As mentioned above, melatonin is secreted in large quantities at night according to its circadian rhythm. Studies illustrates that the nocturnal secretion of melatonin in patients with CHD decreases significantly compared with healthy people and it may be associated with various disease risk factors in these patients which can explain why AMI always happen (at a peak) in early morning when melatonin levels are adequate (Dominguez-Rodriguez et al., 2006, 2007a).

In terms of clinical disease treatment, most studies on melatonin-induced cardiovascular effect are in phase 2a of clinical trials, and according to literatures, melatonin has a beneficial therapeutic effect on hypertension, atherosclerosis, CHD, and other chronic cardiovascular diseases (Yang et al., 2014; Simko et al., 2016; Baltatu et al., 2019) (Table 1). Since it is an age-related molecule, it declines with age. Because of its high safety profile, it has important potential clinical applications. Clinical trials demonstrate that melatonin significantly reduce the area of myocardial infarction when used in the treatment of STEMI (ST-elevation myocardial infarction) patients after PCI (Dominguez-Rodriguez et al., 2017a). In addition, the use of melatonin before surgery significantly reduces CABG related oxidative stress and cardiac injury, and on the other hand increase the activity of Nrf2 in patients (Haghjooy Javanmard et al., 2013; Shafiei et al., 2018). Melatonin plays a role in reducing nocturnal hypertension in patients by affecting circadian cardiovascular rhythms of blood pressure (Grossman et al., 2011). The experimental results of Ma et al. (2018) show that melatonin eliminates ROS by regulating the mitophagy in macrophages, thus inhibiting the activation of NLRP3 (nucleotide-binding domain and leucine-rich repeat pyrin domain containing 3) inflammasome and ultimately inhibiting the progression of atherosclerosis, which is mediated at least in part through the Sirt3 signaling pathway.

**TABLE 1:** A summary of the results of some clinical trials (there are many more), which illustrate the beneficial effects of melatonin in clinical acute myocardial infarction.

Type of study	Study population	Administration route	Results	Possible mechanism
Unicenter, randomized, double-blind, parallel-group, placebo-controlled study (Dominguez-Rodriguez et al., 2007a)	272 patients with AMI and be expected to undergo primary angioplasty (PA); melatonin group (*n* = 136), placebo group (*n* = 136).	Patients received a total intravenous melatonin dose of 11.61 mg (approximately 166 μg/kg) or placebo. The temporal distribution of perfusion was: 30 min previous to percutaneous revascularization and remainder doses in a subsequent 120 min (1 h during the angioplasty + 60 min post-intervention).	The infarction size of melatonin group and placebo group was 9.0% and 19.5%, respectively (*P* < 0.05).	The cardiac-protection effect of melatonin was most likely through its direct free radical scavenging activities, indirect antioxidant activity and its ability to increase mitochondrial bioenergetics.
Case-control study (Dominguez-Rodriguez et al., 2008)	90 patients with STEMI and 70 healthy humans.	No melatonin was administered.	Melatonin value kept adiurnal variation but with a significantly lower dose in STEMI patients (*P* < 0.001). The mean nocturnal melatonin levels in these patients was lower than in the control group (*P* < 0.001).	The lower melatonin production rate in AMI patients was correlated with the stage of the disease, and some immunological factors, such as CRP and cytokines, could play an important role in the pathogenesis.
Prospective cohort study (Dominguez-Rodriguez et al., 2010a)	180 patients with first STEMI who underwent PCI within 6 h from onset of symptoms. 63 patients (35%) were angiographic no-reflow after PCI.	No melatonin was administered.	Patients with angiographic no-reflow had lower intraplatelet melatonin levels compared to patients without no-reflow (12.32 ± 3.64 vs. 18.62 ± 3.88 ng/100,000 platelets, *P* < 0.0001)	Platelets have Melatonin inhibits platelet cyclooxygenase and decreases arachidonic acid-induced aggregation and thromboxane B2 production and thus inhibits platelet aggregation.
Prospective cohort study (Dominguez-Rodriguez et al., 2012)	161 patients with AMI.	No melatonin was administered.	Melatonin levels (OR = 2.10, CI 95% 1.547–2.870, *P* < 0.001) were an independent predictor of LV remodeling.	The anti-fibrotic and antioxidant effect of melatonin.
Nested case-control study (McMullan et al., 2017)	209 women with incident cases of fatal and non-fatal MI and were matched to 209 controls.	No melatonin was administered.	Lower melatonin secretion was significantly associated with a higher risk of MI. Women in the highest concentration had an estimated absolute risk of MI of 84 cases per 100,000 person-years compared with 197 cases per 100,000 person-years in the lowest concentration, and the association was strongly modified by body mass index (BMI) (*p* = 0.02).	Melatonin reduces platelet aggregation, against plaque rupture, and regulates the immune system and inflammation.
Prospective, multicenter, randomized, double blind, placebo-controlled study (Dominguez-Rodriguez et al., 2017b)	146 patients with STEMI; melatonin group (*n* = 73), placebo group (*n* = 73).	The experimental drug was a formulation of melatonin in polyethylene glycol solution. Patients in the melatonin group received a dose of 51.7 μmol intravenously given by a time period of 60 min starting immediately before PCI and a bolus of 8.6 μmol of intracoronary melatonin given through the PCI-guiding catheter after restoring the blood flow to the infarct related artery. The placebo group received a matching placebo formulation.	No significant differences in the myocardial infarct size between the two group. Both left ventricular end-diastolic and end-systolic volumes were lower in the placebo group (*P* = 0.01). No significant differences in the incidence of adverse events at 1 year in both groups (*P* = 0.150).	The median pain-to-balloon time (200 min) was so long that it likely negated the benefits of melatonin in reducing lethal IRI.
Unicenter, randomized, double-blinded, placebo controlled (Ekeloef et al., 2017)	48 patients with STEIMI; melatonin group (*n* = 24), placebo group (*n* = 24).	Patients were randomized to receive either intracoronary or intravenous melatonin (total 50 mg) or placebo (isotonic saline)	Melatonin did not exert a significant effect on myocardial salvage index after PCI. The myocardial salvage index at day 4 (±1 day) after PCI was similar in the melatonin group (*n* = 22) at 55.3% (95% CI 47.0–63.6) and the placebo group (*n* = 19) at 61.5% (95% CI 57.5–65.5), *p* = 0.21.	The cardioprotective effects of melatonin might be largely dependent on a clinically effective distribution of the drug in the myocardial area at risk, prior to ischemia and definitely prior to reperfusion
Prospective, multicenter, randomized, double blind, placebo-controlled study (Dominguez-Rodriguez et al., 2017a)	146 patients with STEMI; melatonin group (*n* = 73), placebo group (*n* = 73). Randomized patients were divided into tertiles according to symptoms onset to balloon time: first tertile (136 ± 23 min), second tertile (196 ± 19 min), and third tertile (249 ± 41 min).	The experimental drug was a formulation of melatonin in polyethylene glycol solution. Patients in the melatonin group received a dose of 51.7 μmol intravenously given by a time period of 60 min starting immediately before PCI and a bolus of 8.6 μmol of intracoronary melatonin given through the PCI-guiding catheter after restoring the blood flow to the infarct related artery. The placebo group received a matching placebo formulation.	In the first tertile, the infarct size was significantly smaller in the melatonin-treated subjects compared with placebo (14.6 ± 14.2 vs. 24.9 ± 9.0%; *P* = 0.003). Treatment with melatonin was associated with a larger infarct size in the group of patients included in the third tertile (20.5 ± 8.7% vs. 11.2 ± 5.2%; *P* = 0.001), resulting in a significant interaction (*P* = 0.001).	Melatonin administered earlier may result in a greater cardioprotective effect compared with delayed administration. Treatments that are able to reduce mitochondrial dysfunction appear to be more effective after shorter ischemic periods.

*AMI, acute myocardial infarction; PCI, percutaneous coronary intervention; STEMI, ST-segment elevation myocardial infarction.*

Studies reveal that (Dominguez-Rodriguez et al., 2017a), from the onset of myocardial infarction symptoms to the beginning of interventional therapy, the application of melatonin in a short time window can effectively reduce the size of a myocardial infarct. Repair after a myocardial infarction is a complex process involved with multiple factors, but whether melatonin is involved with these repair processes have not been investigated. The combination of melatonin with other myocardial protective drugs (e.g., antithrombotic drugs and new-type myocardial metabolic regulation drug GLP1) has not yet been reported, and further research is required to confirm the myocardial protective actions of melatonin treatment.

## Author Contributions

ZF conceived and designed the review. ZF, YJ, JW, YZ, and MS collected the literatures. YJ and ZF wrote the manuscript. ZF, RR, and YC reviewed and edited the manuscript. QX revised the manuscript and the language. All authors read and approved the manuscript.

## Conflict of Interest

The authors declare that the research was conducted in the absence of any commercial or financial relationships that could be construed as a potential conflict of interest.
